# The Physical Activity-Dependent Hematological and Biochemical Changes in School Horses in Comparison to Blood Profiles in Endurance and Race Horses

**DOI:** 10.3390/ani11041128

**Published:** 2021-04-14

**Authors:** Małgorzata Maśko, Małgorzata Domino, Tomasz Jasiński, Olga Witkowska-Piłaszewicz

**Affiliations:** 1 Department of Animal Breeding, Institute of Animal Science, Warsaw University of Life Sciences, 02-787 Warsaw, Poland; malgorzata_masko@sggw.edu.pl; 2 Department of Large Animal Diseases and Clinic, Institute of Veterinary Medicine, Warsaw University of Life Sciences, 02-787 Warsaw, Poland; tomasz_jasinski@sggw.edu.pl; 3 Department of Pathology and Veterinary Diagnostics, Institute of Veterinary Medicine, Warsaw University of Life Science, 02-787 Warsaw, Poland

**Keywords:** physiological demands, effort, exercise, equine, athletes, monitoring

## Abstract

**Simple Summary:**

Leisure horse riding is a leading branch of the equine industry worldwide, thus, horses are used for pleasure and in riding schools represent a much larger group than sports horses. Nevertheless, still little is known about the physiological demands and the nature of the exercise metabolism in school horses. The exercise capacity in sport horses is routinely monitored using blood tests including hemogram parameters and selected biochemical indicators. This study aimed to measure the physical activity-dependent blood indicators during the leisure type of work in riding schools in order to compare them with endurance and race horses’ effort-dependent profiles. The similarities between school and race horses in a high level of erythrogram parameters after effort as well as between school and endurance horses in a high level of white blood cell count and high activity of creatine phosphokinase after effort were demonstrated. However, the fluctuations in physical activity-dependent blood indicators were lower in school than in professional equine athletes. The exception was the lactic acid profile, which achieved higher values than in endurance horses and lower than in race horses. Limiting the school horses monitoring to only the endurance or racing blood profile may result in the omission of significant changes in hematological and biochemical parameters.

**Abstract:**

Blood testing is one of the most important ways to improve performance, facilitate recovery and monitor the training of endurance and race horses. However, little is known about the physical activity-dependent changes of blood parameters in horses used for pleasure and in riding schools. This study aimed to perform routine blood tests for training monitoring of sport horses in three different horse types of use. Then the values of blood indicators were compared between school, endurance and race horses to find similarities in the physical activity-dependent profile. The study was carried out on 15 endurance, 15 race and 15 school healthy horses who underwent the typical effort for their disciplines. The hemogram parameters, creatine phosphokinase (CPK), aspartate aminotransferase (AST), blood lactate (LAC), and total serum protein (TSP) concentrations were measured using the same protocol and equipment. Measurements of main hematological and biochemical physical activity-dependent parameters were conducted before, immediately after and 30 min after training. In school horses, the physical activity-dependent increase of WBC (40.9%) and CPK (76.4%) was similar to endurance horses, whereas an increase of RBC (19.1%), HGB (18.6%) and HCT (19.4%) were more similar to race horses. The moderate effort-dependent increase of LAC concentration (2775%) was lower than in race horses (7526%) and higher than in endurance horses (390%). Limiting the training or work monitoring assessment of school horses to only the endurance or racing blood profile may result in the omission of significant changes in hematological and biochemical parameters.

## 1. Introduction

The clinical examination provided by the medical practitioner and the trainer’s opinion does not always indicate the performance ability of the sportsman. Thus, blood measurements and advanced exercise tests are routinely used to assess several exercise capacity parameters of the human athlete [1]. However, considering equine athletes, significant limitations resulting from invasiveness or requirement of equipment that cannot be transported to the stable restricted the performance of advanced exercise tests on a large population of horses [2]. Therefore, in the case of equine athletes, regular blood sampling was confirmed as useful for detecting equine exercise capacity to improve performance, facilitate recovery, and monitor training [1,2,3]. In veterinary medicine, it is especially important because the fast detection of the low performance is altered by the inability to perform the anamnesis with the patient, as it is in human medicine. The attention is paid mostly to obtain the values of blood parameters such as red blood cell count (RBC), hemoglobin concentration (HGB), hematocrit (HCT), white blood cell count (WBC), lactate concentration (LAC), total serum protein (TSP) concentration, creatine phosphokinase (CPK) and aspartate aminotransferase (AST) activity to monitor the health status and training progress of the sport horse depending on the discipline [2,3,4].

The evaluation of the performance using hematological and biochemical blood indicators requires consideration of the nature of the exercise metabolism in horses in various disciplines. During low-intensity exercise, all energy requirements are met by aerobic mechanisms [5,6,7]. While, physical activity requiring sustained high-intensity performance influences an increased anaerobic energy consumption [2,8]. The example of a low-intensity long-distance physical activity in horses is endurance [3,7]. Endurance effort is an exercise of aerobic nature because 95% of total energy costs are contributed by oxidative phosphorylation of adenosine triphosphate (ATP) [6]. Thus, well-performed endurance horses should be characterized by high aerobic power. Endurance horses accumulate less lactate, thus the lactate threshold is reached only as a result of a fast finish and it is usually not higher than 10 mmol/L [6]. The evaluation of LAC blood concentration in the endurance horse monitoring is therefore replaced by CPK and AST activity. An increased CPK and AST activity confirm muscle fatigue after controlled long-distance races. Increased aerobic power may also be assessed based on hemogram values [2,3]. On the contrary to endurance effort, horse racing represents a typical high-intensity short-duration effort [2,4,6]. During horse racing, the fast glycolytic pathways are recruited and the energy demands increase rapidly depending on the intensity of the race training program [2,3,4,5]. As exercise intensity increases the lactate concentration in the muscle and blood rises, thus, the well-performing race horses should be characterized by anaerobic power [2,8]. Race horses accumulate a lot of lactate, thus, the lactate threshold is usually significantly exceeded [8]. Thus, the LAC concentration is the most important blood parameter measured during race horse performance capacity monitoring. Muscle fatigue may also be confirmed based on hemogram values and CPK and AST activity [2,3]. Since different sport horse types of use are routinely monitored using different physical activity-dependent blood indicators, the assessment of the suitability of blood tests for leisure work in riding schools monitoring is required.

Leisure horse riding is a leading branch of the equine industry worldwide, thus, horses are used for pleasure and in riding schools represent a much larger group than sports horses [9]. The typical effort of leisure horses is variable from horses ridden once a week for pleasure to horses ridden 6 days a week in riding schools [9,10,11,12]. Since still little is known about the physiological demands and the nature of the exercise metabolism in leisure horses, this research focuses on the larger workload group of leisure horses, namely the school horses. For this purpose, the representative group of school horses, that worked 1–2 h a day, 5 days a week under the rider with standardized body weight and upper-intermediate skills, were examined using the standard sport horse monitoring protocol. Recently a few studies on the effort of riding school horses compared only changes in basal hemogram [10,11]. Most of the research in school horses has focused on risk factors associated with health disorders [12,13,14,15,16], welfare state [17] and the aggravating impact of an excessive load of a rider’s body weight [18,19,20]. Among the health disorders of school horses, back pain [12,13], lameness [14,15], and obesity [16] were the most frequently reported. Back pain was also indicated as a consequence of overload during leisure work in riding schools when the rider’s body weight is too high [13,19,20]. Moreover, school horses, unlike endurance or racing horses, are not matched with one rider with high skills. School horses work with many different riders, often beginners, still learning how to sit properly [21], which may cause an increase in physical activity and muscle micro-injuries [22,23]. Regardless of whether the rider is appropriately matched to the horses or the inexperienced rider is not balanced, the blood hematological and biochemical changes provide valuable information about the exercise capacity of horses that successively perform the leisure type of work in the riding school. Without the knowledge on the nature of school horses’ effort under standardized work, the physical activity-dependent accumulation of LAC, fluctuations of CPK and AST activity and changes in hemogram values are difficult to interpret in the case of disorders.

Thus, this study aimed to perform routine blood tests for training monitoring of sport horses in three different horse types of use. Then the values of blood indicators were compared between school, endurance and race horses to find similarities in the physical activity-dependent profile. The hemogram parameters, CPK and AST activities, as well as LAC and TSP concentrations, were measured concerning a typical effort for each discipline, and then compared before and after the training session.

## 2. Materials and Methods

### 2.1. Horses

The study involved 45 healthy horses belonging to three groups: endurance horses (*n* = 15), race horses (*n* = 15) and school horses (*n* = 15). Each horse had been already used for this type of work not less than 1 year.

Fifteen privately-owned, 6–12 years old, endurance Arabian horses (5 mares and 10 geldings) were enrolled in the endurance group. The horses were housed in two stables in similar conditions and trained according to similar protocols. All exercises were provided under similar terrain conditions. The training involved daily sessions with the exercise-load depending on the horse’s condition and increasing with time; altogether each horse covered about 200–300 km per month and the sessions with high exercise-load were performed every 14–20 days.

Fifteen privately-owned, 3–5 years old, race Arabian horses (8 mares, 3 geldings and 4 stallions) were included in the race group. The horses were kept and trained at the same racing track in similar conditions and trained by one trainer. The training involved daily sessions including galloping on the sand for 1000 m. The sessions with high exercise-speed were performed every 2 days in cycles of 5 days a week.

Fifteen WULS-owned, 6–10 years old, school Polish warmblood breeds horses (6 mares and 9 geldings) were involved in the school group. The horses were housed with the same management in the Didactic Stable of Horse Breeding Division at WULS (Warsaw University of Life Sciences). All horses were in daily leisure use in the riding school, namely recreational riding 1–2 h a day, 5 days a week.

Each horse of all three investigated groups received an individually calculated ration of hay, oats and concentrate according to its nutritional requirements arising from maintenance and workload. This diet was distributed over three feedings per day. A mineral salt block and fresh water were constantly available. According to the owners, all horses were dewormed and vaccinated at similar times and did not receive medications or suffer from infection in the preceding 3 weeks. Before and after each training, the basic clinical examinations were conducted. The basic clinical examination including evaluation of internal temperature, heart rate, respiratory rate, mucous membranes, capillary refill time, lymph nodes and was carried out following international veterinary standards. The detailed examination of the musculoskeletal system was performed following the guidelines for the lameness evaluation of the athletic horse [23].

### 2.2. Training Sessions

Three training sessions were designed according to the nature of the exercise metabolism in horses in various disciplines and included endurance training sessions for endurance horses, race training sessions for race horses and leisure training sessions for school horses. In order to limit the impact of additional factors, the riders’ body weight (61.2 ± 2.2 kg) and skills (upper-intermediate) were standardized. Each rider rides regularly at least three times a week and not less than 4 years and achieved the level with a firm seat, is confident and in control at all paces, including rising trot, two-point canters and gallops.

The endurance training sessions included an effort individually adjusted to each horse. The training session selected for the study was the one with a high exercise-load with 25–42 km, speed 3.3–4.5 m/s depending on the trainer’s decision.

The training session in the race horses included about 10 min of walking as a warm-up, 10 min of trot or canter and then gallop over the distance of 1000 m, at a speed of 8–12 m/s, according to horse performance and the trainer’s decision. After that, the horses were cooled down on an automatic horse walker for 30 min.

The leisure training sessions included an effort individually adjusted to each horse for the appropriate tempo: walk (up to 1.5 m/s), trot (up to 4.0 m/s) and canter (up to 6.0 m/s). The leisure training was performed on the indoor-ridden arena on both sides. The total duration of each session was 60 min: 10 min of walk, 10 min of trot, 5 min of walk, 5 min of trot, 5 min of canter, 5 min of trot, 5 min of walk, 5 min of canter, 5 min of trot, and finally 5 min of walk.

### 2.3. Blood Sampling

Blood samples (BS) from race and school horses were collected before training (BS 0), immediately after training (1–3 min; BS 1) and 30 min after training (BS 2). BS 2 were not collected from endurance horses. In all cases BS 0 were collected in the morning, before feeding. Samples collected from endurance and race horses were a part of standard veterinary diagnostic procedures according to Polish legal regulations (art 1.2 (5) Ust. z dnia 15 stycznia 2015 r. o ochronie zwierzat wykorzystywanych do celów naukowych lub edukacyjnych, Dz.U.2018.0.1207 (Resolution on the animals protection used for scientific and educational purposes); the European directive EU/2010/63 approval of the Local Commission for Ethics in Animal Experiments was not required. Blood samples from school horses were collected according to a protocol approved by the II Local Ethical Committee on Animal Testing in Warsaw (Permit Number: WAW2/059/2018, from 27 April 2018) on behalf of the National Ethical Committees on Animal Testing. All blood samples were acquired by jugular venipuncture using a BD Vacutainer^®^ system into K2-EDTA tubes for hematological tests and dry tubes for biochemical analyses (Plymouth, UK).

### 2.4. Hematology and Biochemical Analysis

EDTA blood samples were kept at +4 °C and examined within 5 h in an automated analyzer calibrated for equine species (ABC Vet, Horiba ABX). The following hematological parameters were taken into account: white blood cell count (WBC, ×10^9^/L), red blood cell count (RBC, ×10^12^/L), hemoglobin concentration (HGB, mmol/L), hematocrit (HCT, %), mean corpuscular volume (MCV, fL), mean corpuscular hemoglobin (MCH, gL), mean corpuscular hemoglobin concentration (MCHC, mmol/L), and red cell distribution width (RDW, %).

Blood lactate concentrations (LAC, mmol/L) were determined in whole blood immediately after blood collection using an Accusport^®^ (Roche Diagnostics, Basel, Switzerland). The dry tubes were centrifuged (2000× *g*, 5 min) and serum-free from any apparent hemolysis was aspirated for further analyses. Total serum protein (TSP, g/L) concentration was measured by the refractometer technique (Reichert Rhino Vet 360, Munich, Germany). Creatine phosphokinase (CPK, U/L) and aspartate aminotransferase (AST, U/L) activities were determined using an automated clinical biochemistry analyzer (Miura One, ISE. S.r.l., Albuccione, Italy). For all measurements, Pointe Scientific (5449 Research Dr, Canton, MI 48188, USA) reagents, standards, calibrators, and controls were used.

### 2.5. Data Analysis

Obtained hematological and biochemical data were presented in a form of data series in which subsequent horses were represented by corresponding realizations. Each of the 12 hematological and biochemical blood measurements were tested independently. Each data series were tested independently for univariate marginal distributions using a D’Agostino and Pearson omnibus normality test. Non-Gaussian distribution was stated for the following data in BS 0 of endurance horses: WBC, LAC, TSP; BS 0 of race horses: HCT, LAC, CPK, AST; BS 0 of school horses: LAC, TSP; BS 1 of endurance horses: RDW, CPK; BS 1 of race horses: MCH; BS 1 of school horses: HGB, MCH, RDW, CPK; BS 2 of race horses: HCT, MCH, AST; and BS 2 of school horses: MCHC, LAC, TSP, CPK.

To estimate the differences between measurements (0, 1) of endurance horses, the non-Gaussian distributed data series were compared using Wilcoxon matched-pairs signed rank test (for WBC, RDW, LAC, TSP, and CPK). To estimate the differences between measurements (0, 1, 2) of race and school horses, the non-Gaussian distributed data series were compared using Friedman test with Dunn’s multiple comparisons test (for HCT, MCH, LAC, CPK, and AST in race horses, and for HGB, MCH, MCHC, RDW, LAC, TSP, and CPK in school horses). The multiple comparisons test was used to compare the mean of each data series with the mean of every other data series, independently for each blood parameter.

Other data series, which showed a normal distribution, were compared using paired t test or repeated measures ANOVA summary with Tukey’s multiple comparisons test. The differences between measurements (0, 1) of endurance horses were estimated using paired t test (for RBC, HGB, HCT, MCV, MCH, MCHC, and AST). The differences between measurements (0, 1, 2) were estimated using repeated measures ANOVA summary with Tukey’s multiple comparisons test (for WBC, RBC, HGB, MCV, MCHC, RDW, and TSP in race horses, and for WBC, RBC, HCT, MCV, and AST in school horses). The multiple comparisons test was used to compare the mean of each data series with the mean of every other data series, independently for each blood parameter.

If the biochemical measurements differed significantly between measurements (0, 1 or 0, 1, 2), the percentage difference was calculated as the ratio of the difference between the mean value of BS 0 and mean value of BS 1 or mean value of BS 0 and mean value of BS 2 to the mean value of BS 0, expressed as a percentage. If the biochemical parameters did not differ significantly between measurements (0, 1 or 0, 1, 2), the percentage difference was not calculated. The percentage differences were also not calculated for the hematological measurements.

All results were reported on the figures as mean +SD. All statistical analysis was performed using GraphPad Prism6 software (GraphPad Software Inc., 2365 Northside Dr. Suite 560 San Diego, CA 92108, USA), where the significance level was established as *p* < 0.05.

## 3. Results

In all horses, hematological and serum biochemical parameters determined before and after training fell within relevant reference intervals characteristic for selected groups of horses [4]. All horses at days of BS collections were free from clinical symptoms of diseases. No symptoms of the disease were found in the basic clinical examinations and the detailed examination of the musculoskeletal system both before and after training.

**Figure 1:**
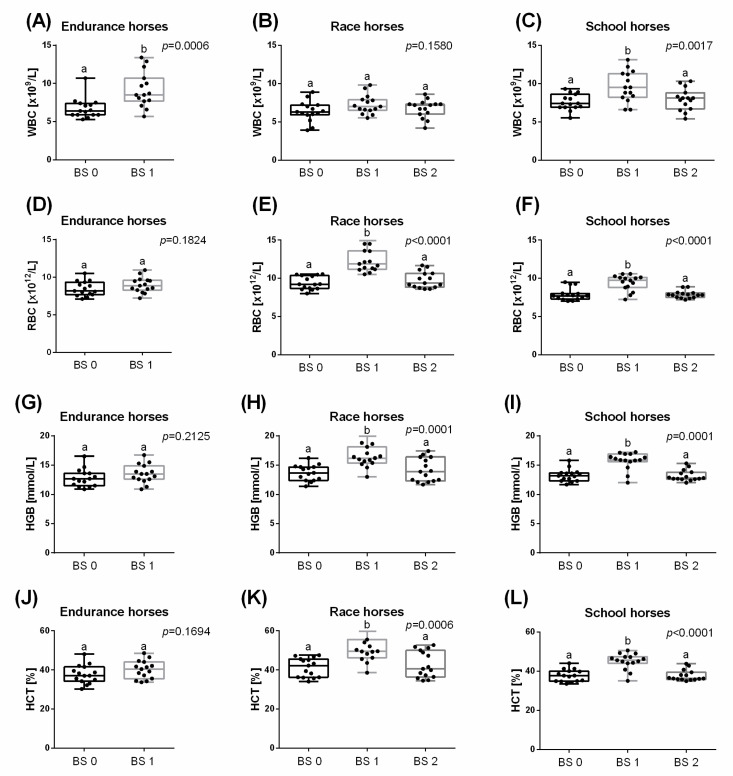
Selected hematological parameters (mean + SD), white blood cell count (WBC) (**A**–**C**); red blood cell count (RBC); (**D**–**F**); hemoglobin concentration (HGB); (**G**–**I**); hematocrit (HCT); (**J**–**L**) measured before training (BS 0; individual values marked with dots), immediately after training (BS 1; individual values marked with squares) and 30 min after training (BS 2; individual values marked with triangles) in endurance horses (**A**,**D**,**G**,**J**), race horses (**B**,**E**,**H**,**K**) and school horses (**C**,**F**,**I**,**L**). Lower case letters indicate differences between BSs for *p* < 0.05.

**Figure 2:**
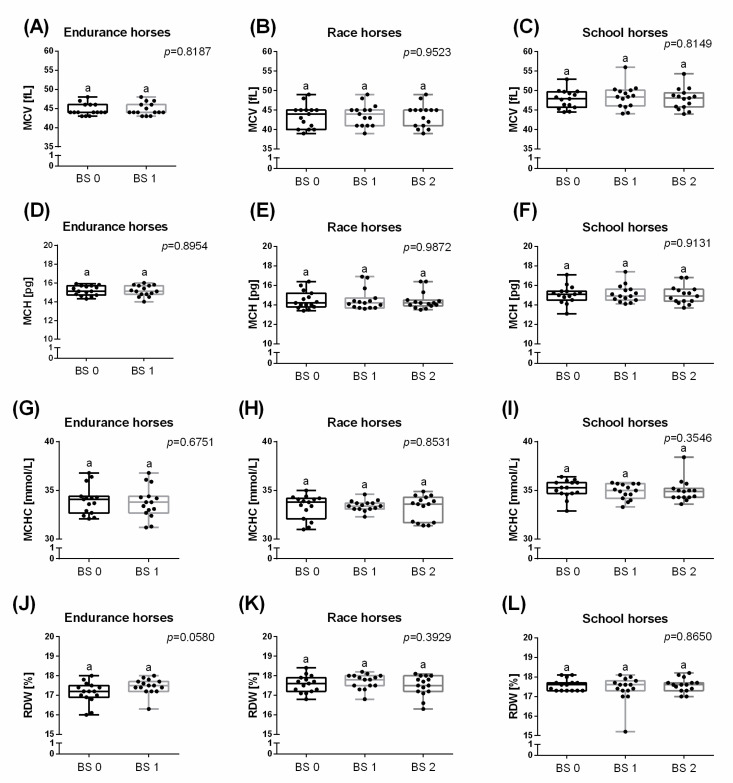
Selected hematological parameters (mean + SD), mean corpuscular volume (MCV) (**A**–**C**); mean corpuscular hemoglobin (MCH) (**D**–**F**); mean corpuscular hemoglobin concentration (MCHC) (**G**–**I**); red cell distribution width (RDW) (**J**–**L**), measured before training (BS 0; individual values marked with dots), immediately after training (BS 1; individual values marked with squares) and 30 min after training (BS 2; individual values marked with triangles) in endurance horses (**A**,**D**,**G**,**J**), race horses (**B**,**E**,**H**,**K**) and school horses (**C**,**F**,**I**,**L**). Lower case letters indicate differences between BSs for *p* < 0.05.

A significant increase for WBC was observed immediately after effort in endurance (36.0%; *p* = 0.0006) and school (40.9%; *p* = 0.0017) horses, but not in race horses. In school horses, WBC decreased to the resting value within 30 min after effort (Figure 1A–C). significant increases for RBC, HGB and HCT were noted immediately after effort in race (RBC: 31.7%; *p* < 0.0001; HGB: 20.6%; *p* = 0.0001; HCT: 24.2%; *p* = 0.0006) and school (RBC: 19.1%; *p* < 0.0001; HGB: 18.6%; *p* = 0.0001; HCT: 19.4%; *p* < 0.0001) horses, but not in endurance horses (Figure 1D,G,J). In both race and school horses, RBC (Figure 1E,F), HGB (Figure 1H,I) and HCT (Figure 1K,L) decreased to the resting value within 30 min after effort. All remaining hematological parameters (MCV, MCH, MCHC, and RDW) did not change depending on the effort in all the studied groups of horses (Figure 2).

**Figure 3:**
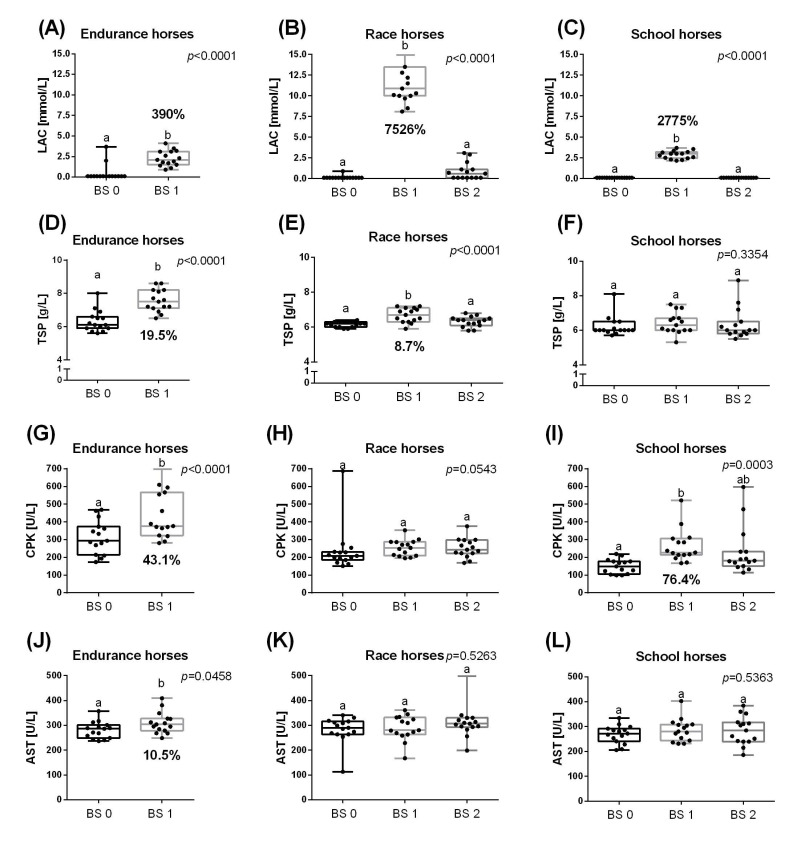
Selected biochemical parameters (mean + SD), lactate concentration (LAC) (**A**–**C**); total serum protein (TSP) (**D**–**F**); creatine phosphokinase (CPK) (**G**–**I**); aspartate aminotransferase (AST) (**J**–**L**), measured before training (BS 0; individual values marked with dots), immediately after training (BS 1; individual values marked with squares) and 30 min after training (BS 2; individual values marked with triangles) in endurance horses (**A**,**D**,**G**,**J**), race horses (**B**,**E**,**H**,**K**) and school horses (**C**,**F**,**I**,**L**). Lower case letters indicate differences between BSs for *p* < 0.05.

The LAC increased significantly immediately after the effort and then decreased to the resting value in all three groups of horses, although in endurance horses it was an increase of 390% (*p* < 0.0001), in race horses by 7526% (*p* < 0.0001) and in school horses by 2775% (*p* < 0.0001) (Figure 3A–C). The TSP concentration increased significantly immediately after effort in endurance (19.5%; *p* < 0.0001) and race (8.7%; *p* < 0.0001) horses, but not in school horses. In race horses, TSP concentration decreased to the resting value within 30 min after effort (Figure 3D–F). The CPK activity increased immediately after effort in endurance (43.1%; *p* < 0.0001) and school (76.4%; *p* = 0.0003) horses, but not in race horses. In school horses, the decrease of CPK activity lasted longer than 30 min after effort (Figure 3G–I). The AST activity increased after effort only in endurance horses (10.5%; *p* = 0.0458) (Figure 3J–L).

## 4. Discussion

The relationship between hematological and blood biochemical parameters and training has been previously reported [2,4,6]. In some studies, erythrogram values increase within the training period. Increased oxygen transport via RBC leads to higher aerobic capacity. However, some studies failed to demonstrate differences in fitness and blood parameters in horses [2,4,6]. It may be associated with an attitude of the horse and feeding status which have an impact mostly on resting blood parameters.

It is important to be aware that any physical activity is associated with accelerated metabolism. In endurance horses, it was documented that RBC, HGB and HT are not as strongly affected by the physical activity during training as in race horses [3,6]. In the study presented here, the highest increase in erythrogram parameters was noted in race horses (31.7%) in comparison to school horses (19.1%) and lack of a similar increase in endurance horses. Exercise-induced changes in hematological parameters are known to result from spleen contraction where the blood cells are accumulated [24,25]. In horses, the splenic reserves are huge, especially in those trained for the race [24,25]. In race horses, high-intensity short-duration effort engages a different type of metabolism mostly depending on glycolysis [4,6]. While, the low-intensity long-duration effort, in endurance horses, benefits through elevated oxidative phosphorylation [4,6]. During anaerobic conditions, glycolysis is activated. This is the process that does not require oxygen and extracts energy from pyruvates. It leads to the production of two molecules of ATP [26]. In contrast, oxidative phosphorylation generates 26 molecules of ATP, thus, it is highly more effective [27]. In race horses, physical exertion is connected with high energetic demand which leads to a cumulative deficit of oxygen imposed on muscles to sustain increased workload during training. It leads to the creation of the metabolic state of skeletal muscles called the “oxygen debt” [6]. However, to provide better oxygen delivery, because still, it leads to higher ATP production, the sprinter runners have higher splenic reserves [26]. Higher numbers in RBC and HGB concentration allow better transportation of oxygen to working muscles. In this aspect, school horses seem to be more similar to race horses than endurance horses. However, after leisure type of work, the increases of erythrogram parameters were not as high as in race horses.

When the compensatory anaerobic pathway is activated it leads to increased lactate production. The normal value of the basal blood LAC concentration in horses is close to 1 mmol/L [28]. LAC is released to the bloodstream from muscles depending on the type, intensity and duration of the exercise [29]. The lactate itself is an effective energy source in muscle tissue. Besides, exercise-induced adaptations are driven by those molecules by enhancing myogenesis and regulating muscle acidosis [30,31]. Only training at speeds greater than about 8–10 m/s is leading to the creation of the oxygen debt leading to an increase in blood LAC concentration [6,8]. The high exercise-speed effort is thus achieved only during race training, whereas in endurance horses the oxidative phosphorylation plays the dominant role. Therefore, the LAC measurements are only performed in the monitoring of the training process in race horses [4,8] and the question arises whether LAC monitoring could be useful in assessing the effort of school horses. In the study presented here, this group of horses was characterized by a medium increase in blood LAC concentration after exercise (~2700%), lower than in race horses (~7500%) and higher than in endurance ones (~390%). Those changes in blood LAC concentration suggested the mixed nature of aerobic and anaerobic metabolism during effort. Currently, it also was suggested that some horses may work primarily aerobically and then some lactate may be produced in the muscles during certain movements. Such a mixed LAC concentration profile may exist when most of the time during training is spent in a walk and medium to extended trot and canter [32]. School horses perform a similar type of work, however, the exact nature of the physiological demands during leisure training requires further research.

It should be kept in mind that the decrease in plasma volume influences hematological findings after exercise [3,6]. During physical activity, the TPS is increasing due to training-induced hypervolemia [3,24]. The increased blood volume following an effort leads to greater osmolar and thermoregulatory stability, as well as an expansion of vascular volume for greater cardiac filling [4,6]. In the present study in endurance horses, the increase of TPS after training was higher (19.5%) than in race horses (8.7%). It may be connected with a longer duration of the exercise which lasted 3–4 h whereas race training is more or less 1 h duration. TPS concentration was unchanged in school horses, which may be associated with low-intensity and low-duration physical activity. During this type of horses’ work the extensive fluid loss in the sweat, the reduction of renal flow and the muscle damage responsible for the increased protein catabolism seem to be restrained [33].

In horses, the most accepted blood indicators of muscle damage are CPK and AST activities [34,35]. The highest rate of muscle fatigue is observed during endurance type of work, especially in the competitions at the 160 km distances [35,36]. After the strenuous exercise, CPK activity increases 4–35-fold whereas AST activity increases 2–6-fold [3,37]. In the study presented here, CPK activity was slightly elevated, less than 1-fold in endurance and school horses. The highest increase in CPK activity (76.4%) in school horses may be influenced by the poorest conditioning which may attenuate this enzyme activity [3,33]. On the other hand, the AST activity increased only in endurance horses, thus may be connected with the highest muscle fatigue during this type of exercise. Besides, the CPK and AST values are related to muscle mass and composition [38,39], which were not included in this study.

Leukocyte migration is a crucial process in homeostatic conditions [38,39,40]. In the study presented here, the WBC significantly increased after endurance (36.0%) and leisure (40.9%) training. It may be caused by higher cortisol release during this kind of exercise. The redistribution of WBC from the marginal pool is mostly regulated by cortisol release. Following the exercise, depending on its intensity, the cortisol blood level increases with the peak usually 15–30 min after the beginning and reverses to pre-exercise levels within an hour [41,42]. The highest values of this hormone blood concentration are observed especially after endurance effort and they are higher more or less than 30% than following other physical activities [43]. The higher cortisol release is associated with increased aerobic activity, since the cortisol stimulates both glycogenolysis and lipolysis to produce energy from the fatty acids and glycogen in working muscle [44]. An increased WBC immediately after effort in school horses may then be the second indication of mixed metabolism. However, in the present study, the effect of cortisol on effort-dependent WBC redistribution has not been investigated and it requires further research.

The main limitation as an influencing factor on the individual horse performance and activity-dependent hematological and biochemical changes in blood profiles is the different breeds enrolled to the study. However, pedigree analysis showed that some of Polish native horses are of the Arab type [45]. Thus, the power of influence may be quite marginal. Additionally, the blood samples being collected exclusively during a single standard veterinary procedure in race and endurance groups. Thus, in endurance horses we were not able to collect the blood samples in 3-time point procedure. However, in all protocols for monitoring the endurance training there is no extra sampling during recovery period because of the different nature of physical activity as in race horses [4].

## 5. Conclusions

The physiological demands of school horses partially corresponded with both endurance and race horses. Due to the physical activity-dependent changes in the blood indicators which are in line with both endurance and racing horses’ blood profile, the effort of school horses should be monitored comprehensively. The physical activity-dependent similarities with endurance horses’ blood profile included after effort WBC and CPK increase, whereas an increase of RBC, HGB and HCT indicated the similarity to race horses’ blood profile. Changes in blood LAC concentration suggested an occurrence of typical school horses’ blood LAC profile, which achieved higher values than in endurance horses and lower than in race horses. However, more studies are required to determine the exact nature of the predominant school horses’ metabolism, the role of cortisol in WBC redistribution and the clear recommendations for the monitoring of blood parameters in school concerning typical leisure effort in riding schools. Limiting the training or work monitoring assessment of school horses to only the endurance or racing blood profile may result in the omission of significant changes in hematological and biochemical parameters.

## Data Availability

The data presented in this study are available on request from the corresponding author.

## Abbreviations

AST	aspartate aminotransferase
ATP	adenosine triphosphate
BS	blood samples
CPK	creatine phosphokinase
HCT	hematocrit
HGB	hemoglobin concentration
LAC	lactate concentrations
MCH	mean corpuscular hemoglobin
MCHC	mean corpuscular hemoglobin concentration
MCV	mean corpuscular volume
RBC	red blood cell count
RDW	red cell distribution width
SD	standard deviation
TSP	total serum protein
WBC	white blood cell count
WULS	Warsaw University of Life Sciences
